# Diversity and Genetic Relationship of Free-Range Chickens from the Northeast Region of Brazil

**DOI:** 10.3390/ani10101857

**Published:** 2020-10-12

**Authors:** Débora Araújo de Carvalho, Amparo Martínez Martínez, Inês Carolino, Maria Claudene Barros, María Esperanza Camacho Vallejo, Fátima Santos-Silva, Marcos Jacob de Oliveira Almeida, Nuno Carolino, Juan Vicente Delgado Bermejo, José Lindenberg Rocha Sarmento

**Affiliations:** 1 Department of Animal Science, Federal University of Piauí (UFPI), Campus Universitário Ministro Petrônio Portella, 64049-550 Teresina, Piauí, Brazil; 2 Department of Genetics, Faculty of Veterinary Sciences, University of Cordoba, 14071 Cordoba, Spain; amparomartinezuco@gmail.com (A.M.M.); id1debej@uco.es (J.V.D.B.); 3 Animal Breeding Consulting sl., C/. Astrónoma Cecilia Payne, ID-1, 8-PE, Rabanales 21, 14014 Cordoba, Spain; 4 National Institute for Agrarian and Veterinarian Research (INIAV), Fonte Boa, 2005-048 Vale de Santarém, Portugal; ines.carolino@iniav.pt (I.C.); fatima.santossilva@iniav.pt (F.S.-S.); nuno.carolino@iniav.pt (N.C.); 5 Vasco da Gama University School, 197 Lordemão, 3020-210 Coimbra, Portugal; 6 Department of Chemistry and Biology, Laboratory of Genetics and Molecular Biology, State University of Maranhão (UEMA), Center for Higher Studies of Caxias, 65604-380 Caxias, Maranhão, Brazil; mbdene@yahoo.com.br; 7 Andalusian Institute of Agricultural and Fisheries Research and Training (IFAPA), Alameda del Obispo, 14004 Córdoba, Spain; mariae.camacho@juntadeandalucia.es; 8 Brazilian Agricultural Research Corporation (Embrapa) Meio-Norte, Sector of Technology Transfer, Bairro Buenos Aires, 64006-220 Teresina, Piauí, Brazil; marcos.almeida@embrapa.br; 9 Centre for Interdisciplinary Research in Animal Health (CIISA), University of Lisbon, Avenida da Universidade Técnica, 1300-477 Lisbon, Portugal

**Keywords:** conservation, *Gallus gallus*, genetic resources, microsatellites, native breeds

## Abstract

**Simple Summary:**

Local animal breeds represent a national genetic heritage for every country. Creole free-range chickens have important cultural, historical, genetic, and economic roles in several countries. In Brazil, there is a lack of information regarding the genetic formation of local chicken breeds. These animals were brought to Brazil during colonization in the 16th century. Currently, Brazilian Creole chickens are highly adapted to the edaphoclimatic conditions of the country and are mostly reared by smallholders. In this study, we used microsatellite markers to determine the genetic composition of three chicken breeds from the northeast region of Brazil. Our results confirm the existence of interbreed genetic diversity and high genetic variability within the Brazilian Creole chickens studied. Furthermore, our findings show that the formation of these genetic groups had contributions from different ancestors. Our results will be useful to support the development of conservation programs, as well as the sustainable use and official recognition of these breeds.

**Abstract:**

In this study, we aimed to evaluate the genetic diversity within and among chicken breeds from the northeast region of Brazil (states of Bahia and Piauí) using microsatellite markers. In addition, we assessed the identity and genetic relationships of chickens from Europe, Africa, and South America, as well as their influence on the formation of the Brazilian breeds. A total of 25 microsatellite markers and a panel containing 886 samples from 20 breeds (including the Brazilian chickens) were used in this study. Different statistical parameters were used to estimate the genetic diversity and relationship among the genetic groups studied. Our study indicates that the Brazilian Creole chickens have high genetic variability. The results show that chickens reared in the states of Bahia and Piauí could have originated from different ancestors. The Brazilian breeds studied have an evolutionary relationship with chickens from Portugal, Nigeria, Chile, and Spain. Our results will contribute directly to the conservation and recognition of Brazilian Creole chicken breeds and provide a solid basis for the demonstration of their genetic identity and genetic conservation of American Creole chicken populations.

## 1. Introduction

Local animal breeds are considered a national genetic heritage, and thus, they are important resources for the future of every country. There is a great interest in the conservation and sustainable use of those breeds worldwide. The knowledge of the genetic organization of local genetic resources increases their valuation and recognition. Furthermore, this information is valuable for the genetic management and establishment of conservation programs. Thus, the protection of local breeds is the responsibility of each country, as determined by international agreements [1].

In the northeast region of Brazil, there are several genetic groups of free-range chickens, such as the local breeds Canela-Preta (CP), Caneluda do Catolé (CAN), and Peloco (PEL). These animals have a dual purpose (production of meat and eggs) and are mostly raised by smallholders using low or no technological investment, in extensive systems. Birds of the genetic groups mentioned above are less susceptible to diseases, cope better than other chicken breeds in the harsh environment of Northeastern Brazil, and have tolerance to poor nutrition [2,3].

**Figure 1:**
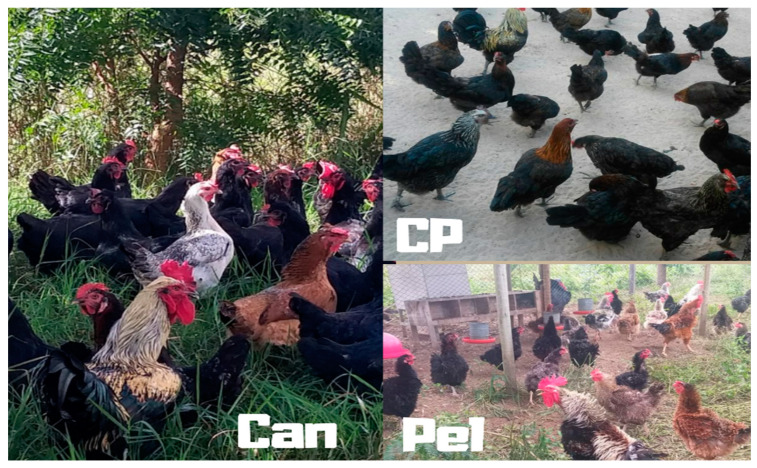
Chicken breeds from the northeast region of Brazil. CP, Canela-Preta; CAN Caneluda do Catolé; PEL, Peloco (Source of pictures: Débora Carvalho and Ronaldo Vasconcelos).

Chickens of the Canela-Preta breed are docile and easy to handle; the plumage of these animals is predominantly black, but they can also present colored feathers around their necks, i.e., white or golden in females, and white, silver, or red in males (Figure 1); their eggs are multicolored, i.e., with brown, yellow, or greenish blue shells. Birds of the Caneluda do Catolé breed are robust, have long legs, a characteristic plumage (their feathers are black and have shades of bluish grey), and lay large eggs. The Peloco chicken breed is characterized by the late feathering during the growing phase and young animals with bristle feathers. Peloco chickens present various plumage colors; they have the appearance of ornamental birds and lay brown, white, red, and greenish blue eggs [2,3].

The genetic structuring and geographical distribution of local breeds can be important to support studies on the dynamics of the human culture, as animals have accompanied human migration for centuries. Thus, knowing the genetic constitution of animal species can provide information that could be historically related to the development of civilizations [4,5]. It is believed that free-range chickens were introduced in Brazil during Portuguese colonization in the 16th century. Subsequently, other small contributions were given by people from other countries, such as Spain (Figure S1) [6].

The individual genetic evaluation of local breeds, as well as the genetic relationship among them and their contextualization regarding other international genetic influences, can provide evidence of mechanisms and events that contributed to the origin and development of free-range chicken breeds in the northeast region of Brazil. A similar study was conducted by Toalombo Vargas et al. [7] in Ecuadorian Creole chickens.

Researchers around the world have conducted studies that aim to shed light on the genetic differentiation of free-range chickens, how the evolution of these animals occurred, and how their diversity can be measured. In this context, microsatellite markers have been widely used to describe the genetic diversity and structure of populations of different domestic species. In general, comparative studies on the genetic diversity and relationship of different breeds consolidate a wide database that can be used for conservation, selection schemes, and understanding of the evolution of species [8].

In order to support projects for the study of the biodiversity of chickens from Ibero-American countries, the BIOCHIKEN consortium (http://www.uco.es/conbiand/consorcios.html) was created within the RED CONBIAND network. This consortium has participated in studies that aim at the genetic characterization of breeds at the national [9] and international [7,10] levels. To the best of our knowledge, previous in-depth studies on the relationship among chickens raised in Northeastern Brazil have never been carried out.

Therefore, this study aimed to evaluate the genetic diversity within and among chicken breeds from the northeast region of Brazil (states of Bahia and Piauí) using microsatellite markers. With this, we aim to provide a basis to support the characterization, national and international recognition, conservation, sustainable use, and valuation of the local chickens. For this purpose, we used a panel containing 25 microsatellite markers to assess the diversity and genetic relationship of three Brazilian chicken breeds. In addition, a panel containing 886 samples from 20 genetic groups (including Brazilian chickens) from various countries previously genotyped was used to determine the genetic identity of breeds and to identify their potential recent influences on the formation of the Brazilian breeds.

## 2. Materials and Methods

### 2.1. Ethics Statement

This study was approved by the Committee on Ethics in the Use of Animals (CEUA) of the Federal University of Piauí, Teresina/ Piauí, Brazil (Nº 399/17).

### 2.2. Sampling

**Figure 2:**
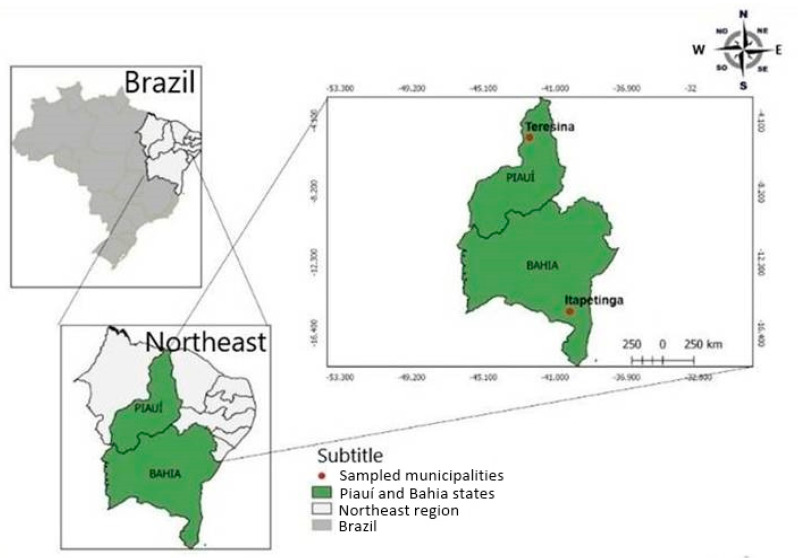
Map showing the geographical locations of the northeast region of Brazil where samples of the three Brazilian chicken breeds (Canela-Preta, Caneluda do Catolé, and Peloco) were collected.

A dataset composed of 100 animals was used to assess the inter- and intrapopulation genetic diversity of free-range chickens from Northeastern Brazil. The local chickens were of three breeds: Canela-Preta (40); Caneluda do Catolé (30); and Peloco (30). The samples were collected randomly from animals raised in conservation nuclei located in Itapetinga/Bahia and Teresina/Piauí, Brazil. Canela-Preta chickens are widely spread throughout the Piauí state, whereas the Caneluda do Catolé and Peloco breeds are widespread in Southwestern Bahia (Figure 2).

In this study, we also used 886 samples of 20 breeds (including Brazilian chickens and a dataset of the BIOCHICKEN research consortium) to determine the possible recent influence of Creole chicken breeds from other countries and commercial strains on the genetic identity of the current Brazilian chicken populations (Table 1). The Creole populations from countries of the Iberian Peninsula (Portugal and Spain) included in the dataset of the BIOCHICKEN research consortium have some influence on the Brazilian breeds.

In addition, Araucana chickens from Chile were used in this study. Moreover, chickens from the north of Africa (Nigeria) represented in the dataset of the BIOCHICKEN research consortium were used. These animals may have some genetic relationship with Brazilian chickens due to the historical period of slavery in Brazil, when people from Nigeria were brought to Brazil and forced to work as slaves. Potential recent influences of the commercial lineages Leghorn and Cornish were also considered, as these strains contributed to the origin of commercial laying and commercial broiler lines, respectively.

### 2.3. DNA Amplification, Molecular Markers, and Genotyping

Fragments of each biological sample were used for extraction of genomic DNA. The procedures used for DNA extraction followed a modification of the method described by Walsh et al. [11]. Three circles were cut in filter papers exposed to a flat surface using a 2 mm Harris Micro punch (GE healthcare Life Science, Little Chalfont, Buckinghamshire, UK), which was cleaned using 1% bleach solution between each sample. The circles were placed in a polymerase chain reaction (PCR) plate and incubated in 100 µL of a 5% CHELEX 100 resin solution (Bio-Rad, Hercules, CA, USA). Subsequently, the PCR plate was incubated in a thermocycler at 95°C for 15 min, 60°C for 15 min, and finally 99°C for 3 min. The lysate was removed and frozen at −20°C until use.

The following 25 microsatellite markers from the AVIANDIV project (http://aviandiv.tzv.fal.de/) and recommended by FAO (Food and Agriculture Organization of the United Nations) [12] were used: ADL112; ADL268; ADL278; LEIO094; LEIO166; MCW016; MCW020; MCW034; MCW037; MCW067; MCW069; MCW078; MCW081; MCW103; MCW104; MCW111; MCW123; MCW165; MCW183; MCW206; MCW216; MCW222; MCW248; MCW295; and MCW330. The amplification of specific fragments of DNA using PCR, as well as electrophoresis conditions were followed as reported in Ceccobelli et al. [13]. Genotypes were read using ABI PRISM GeneScan 3.1.2 (Applied Biosystems, Forster City, CA, USA) and interpreted with ABI PRISM Genotyper 3.7 NT (Applied Biosystems).

### 2.4. Statistical and Genetic Analyses

The program GenAlEx 6.5 [14] was used to estimate the mean number of alleles (NA), the expected heterozygosity (He), and the observed heterozygosity (Ho). The distribution of genetic variability intra- and interbreeds was assessed using Wright’s F-statistics (FIS—Coefficient of inbreeding within individuals, FIT—Coefficient of inbreeding in relation to the total population, and FST—global inbreeding of the population), as well as the matrix of Nei’s genetic distance, the dissimilarity matrix, the principal component analysis (PCoA), and dispersion graph. The PCoA uses the correlation matrix to transform a set of variables Z_1_, Z_2_, …, Z_p_ into a new set of uncorrelated variables Y_1_ (*PC_1_*), Y_2_ (*PC_2_*), …, Y_p_ (*PC_p_*) ordered in decreasing order of variance. The main idea of this procedure is that the first few principal components explain most of the variability in the original data. These estimates were used for the three Brazilian breeds studied.

The global genetic variance among breeds was estimated using analysis of molecular variance (AMOVA) [15]. For this analysis, 10,000 permutations were performed using GenAlEx 6.5 [14] considering the prior clustering of samples organized by breed.

Deviation from the Hardy–Weinberg equilibrium (HWE) at each locus within populations was tested using GENEPOP v.4.0.10 [16].

A neighbor-net was constructed as implemented in the SplitsTree4 software, in order to represent the relationships between breeds graphically and to depict any evidence of admixture [17].

The STRUCTURE software v.2.3.4 [18] was used to determine the most likely number of groups (K) using Bayesian methods with prior information on the origin of samples. We used 1,200,000 simulations of Markov chain Monte Carlo with burn-in of 400,000, admixture model ancestry, with prior information, and tested values of K ranging from 2 to 22, with 10 iterations for analyses of the 20 genetic groups (18 native breeds and two commercial lines). The most likely number of K was calculated using the STRUCTURE HARVESTER web server [19] using the Delta K values according to the method described by Evanno et al. [20], as follows: ΔK = mΔL’(K + 1) − L’(K)/s[L(K)], where L: L’(K) = L(K) − L(K − 1)/s[L(K)]; m represents the mean; s is the standard deviation; and K is the number of proposed groups.

The CLUMPAK program [21] was used to assess the stability among the 10 simulations for the most likely number of K.

## 3. Results

### 3.1. Inter- and Intrabreed Genetic Diversity of the Three Populations of Chickens from the Brazilian Northeast

The mean number of alleles by breed was 4.960 for Canela-Preta (CP) and Peloco (PEL), and 5.040 for Caneluda do Catolé (CAN) (Table 2). The average values of genetic variability measured by Ho for the Brazilian chickens were 0.617 (CP), 0.642 (CAN), and 0.646 (PEL), while the estimates of He were 0.618, 0.634, and 0.630 for CP, CAN, and PEL, respectively. Regarding the genetic differentiation, the three breeds showed negative values for Wright’s fixation index (F). No significant deviations (*p* > 0.05) from the HWE were observed in the Brazilian chickens.

The AMOVA indicates that the highest genetic variability (89%) is distributed within breeds/individuals (Table 3). The variance between populations/breeds represented 8% of the total variation. The Wright’s F-statistics used to assess the population structure indicate that the chickens studied compose genetic subsets equivalent to their own breed, with moderate genetic differentiation (<0.05), as observed in the FST value (0.082). The FIT coefficient (global inbreeding of the population) was 0.108 and the FIS value was 0.029.

### 3.2. Genetic Relationships between Brazilian and Exotic Chicken Breeds

**Figure 3:**
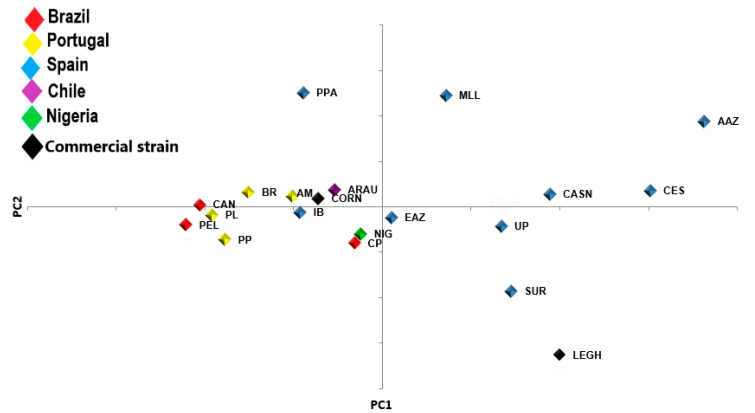
Graphical dispersion of inter-population distances of the 20 genetic groups of chickens in relation to the Cartesian axes obtained by the principal components (PC_1_ and PC_2_) based on the dissimilarity matrix. CP, Canela-Preta; CAN, Caneluda do Catolé; PEL, Peloco; AAZ, Andaluza Azul; CASN, Castellana Negra; CES, Combatiente Español; EAZ, Extremeña Azul; IB, Ibicenca; MLL, Mallorquina; PPA, Pita Pinta; SUR, Sureña; UP, Utrerana Perdiz; ARAU, Araucana; NIG, Nigerian chicken; AM, Amarela; BR, Branca; PLU, Preta Lusitânica; PP, Pedrês Portuguesa; LEGH, Leghorn; CORN, Cornish.

The results of the principal component (PCoA) analysis (Figure 3) demonstrates that the two first PCs explained 34.63% of the genetic diversity of the dataset evaluated. The first axis of the graph comprised the breeds from Brazil, Portugal, Nigeria, and Chile, two Spanish genetic groups, and the Cornish commercial strain. The second PC was composed of seven Spanish breeds and the Leghorn commercial lineage.

The matrix of Nei’s genetic distance considering all genetic groups studied (Table S1) showed that the Nigerian chickens (NIG) were the closest genetically to the CP breed from the state of Piauí, Brazil. The breeds from the Brazilian state of Bahia, i.e., CAN and PEL, were similar (0.063). CAN was close to the Araucana (ARAU) chickens from Chile (0.170), and PEL was close to the Portuguese breed Preta Lusitânica (PLU) (0.156).

**Figure 4:**
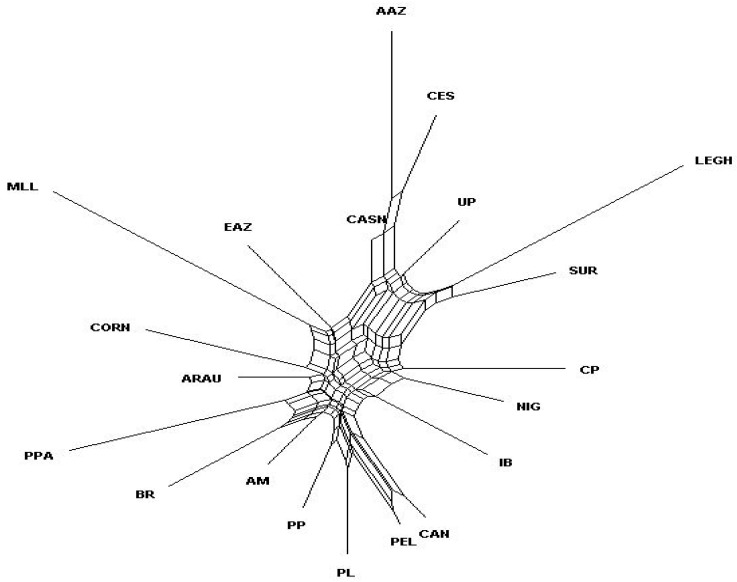
Neighbor-net constructed using the Nei’s genetic distance between the 20 breeds in study. CP, Canela-Preta; CAN, Caneluda do Catolé; PEL, Peloco; AAZ, Andaluza Azul; CASN, Castellana Negra; CES, Combatiente Español; EAZ, Extremeña Azul; IB, Ibicenca; MLL, Mallorquina; PPA, Pita Pinta; SUR, Sureña; UP, Utrerana Perdiz; ARAU, Araucana; NIG, Nigerian chicken; AM, Amarela; BR, Branca; PLU, Preta Lusitânica; PP, Pedrês Portuguesa; LEGH, Leghorn; CORN, Cornish.

The Brazilian breeds were not clustered together with the commercial lines used as control group (Figure 4). Furthermore, the Creole chickens from Brazil were clustered with breeds from Chile, Africa, and Portugal. CP was the only Brazilian genetic group which clustered together with a Spanish breed (Ibicenca).

**Figure 5:**
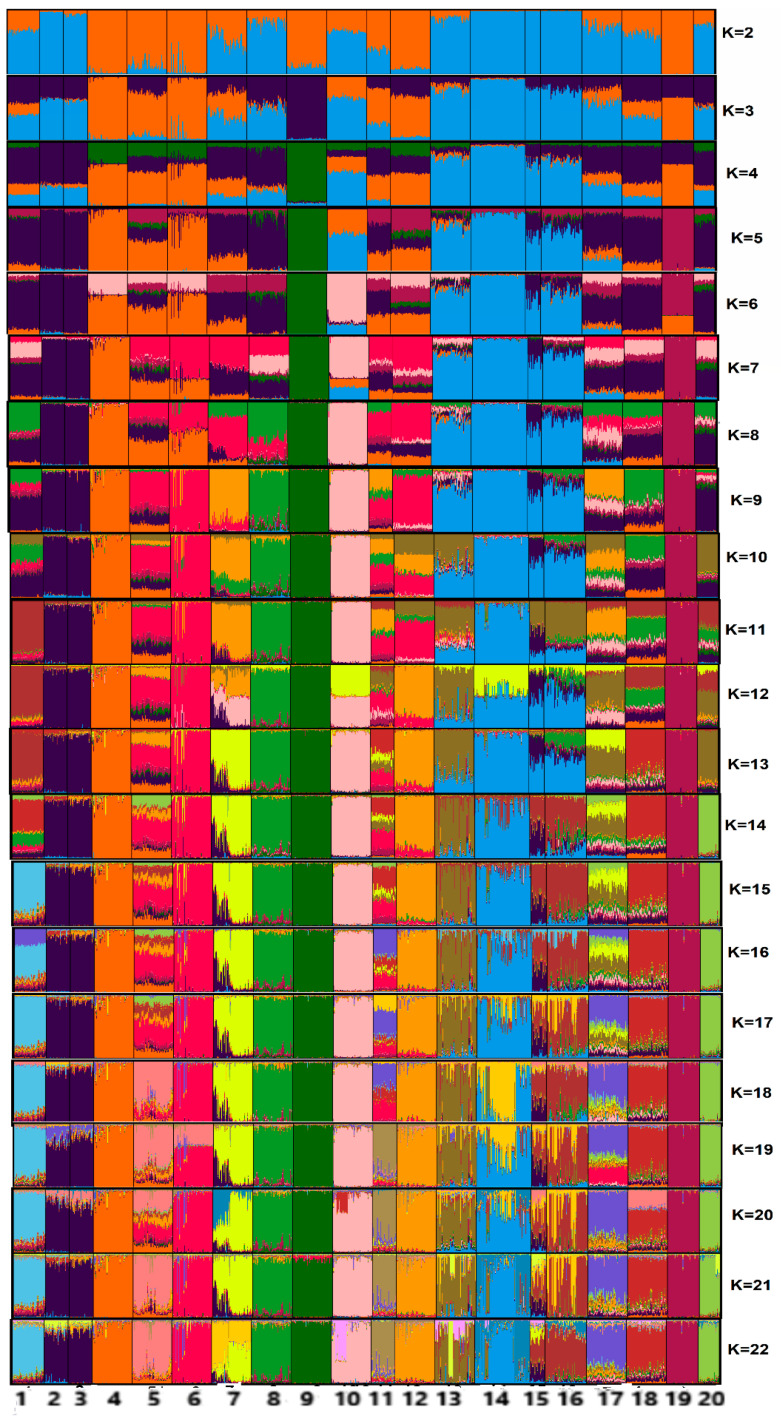
Analysis of population structure using the STRUCTURE software with 886 individuals representing the 20 groups of chicken studied (18 native breeds and two commercial strains) based on 25 microsatellite markers. 1, Canela-Preta; 2, Caneluda do Catolé; 3, Peloco; 4, Andaluza Azul; 5, Castellana Negra; 6, Combatiente Español; 7, Extremeña Azul; 8, Ibicenca; 9, Mallorquina; 10, Pita Pinta; 11, Sureña; 12, Utrerana Perdiz; 13, Amarela; 14, Branca; 15, Preta Lusitânica; 16, Pedrês Portuguesa; 17, Araucana; 18, Nigerian chicken; 19, Leghorn strain; 20, Cornish strain.

In the starting point, we proposed two populations (K = 2) (Figure 5). Under this hypothesis, seven Spanish breeds and the commercial strain Leghorn (LEGH) formed a genetic group. The other group was composed of chickens from Portugal, Brazil, Chile, and Nigeria, the commercial line Cornish (CORN), and two breeds from Spain. The population structure based on the Bayesian method indicated the highest mean value of Delta K (K = 5) (Figure S2). In this case, a group was composed of the three Brazilian breeds, ARAU, NIG, two Spanish breeds (Extremeña Azul (EAZ), Ibicenca (IB), and the European commercial strain CORN). The second group comprised chickens from Portugal and the Spanish breed PPA. The third group included only the Mallorquina (MLL) breed, whereas the fourth group was composed of the commercial strain LEGH, and the fifth group included six Spanish breeds. Despite the lower peaks, there is a trend for the formation of two possible groups (K = 13 and K = 18), which highlights the genetic originality of the three Brazilian breeds in relation to the exotic chickens.

## 4. Discussion

### 4.1. Genetic Diversity within and among the Three Populations of Chickens from the Brazilian Northeast

The mean number of alleles (NA) is an indicator of genetic variability that is essential to establish the long-term evolutionary potential of the population. The NA values found in the present study were higher than those reported by Ceccobelli et al. [13] in five local chicken breeds from Italy (2.63 to 3.67 alleles by breed). These authors used the same set of markers used in our study.

The average values of genetic variability measured by the heterozygosity (Ho and He) for the Brazilian chickens indicate that there is equilibrium between Ho and He. This can be due to the fact that these animals are raised on conservation nuclei, where the maximum variability among breeds must be maintained.

We observed a small variation of Ho and He among the three Brazilian breeds. Several studies in chickens have shown the importance of microsatellite markers using population parameters in analyses of genetic diversity and population structure. Information obtained from those markers allows different direct applications that benefit the genetic management of those birds, as well as their conservation and genetic improvement [22,23,24].

The negative values observed for Wright’s fixation indices probably indicate that there is a trend of fixation of the heterozygosity in the populations studied; therefore, it is suggested that the breeds have genetic variability. This result corroborates the nonsignificant deviations from the Hardy–Weinberg equilibrium observed in the three Brazilian breeds. These chickens compose large populations where random mating system is used; therefore, the genetic variability of these animals is important, as this indicates that these native resources are genetically conserved.

The AMOVA indicates that the highest genetic variability is distributed within breeds, i.e., within individuals, which is relevant as these chickens are native and were not still included in any breeding or selection program. Additionally, this indicates that these breeds have high genetic richness, which makes them potential future donors of genes for the development of new commercial strains and indicates their potential for within-breed selection for genetic improvement practices.

The genetic richness of local genetic groups allows one to use the wide range of options aforementioned in future focal point studies. The percentage of variation between breeds (8%) indicates some similarity among these animals, as they belong to the same subspecies (*Gallus gallus domesticus*), and probably have common ancestors. Nevertheless, this value can be considered high when compared to other studies in Brazilian chicken breeds [6,25].

FST values indicate low (from zero to 0.05), moderate (between 0.05 and 0.15), high (from 0.15 to 0.25), and very high (above 0.25) genetic differentiation between populations [26,27]. Our result for FST is considered moderate (0.082). Comparative studies using microsatellite markers reported low FST (0.029) in a Creole chicken breed from Brazil and high FST in five Italian local chicken breeds (0.225) [6,13].

The FIS value obtained in our study (0.029) indicates the existence of genetic variability within breeds, controlled percentage of homozygosity within populations, and random association of alleles. The FIT value (0.108), in turn, may be indicative of the fact that genes derived from a common ancestor were favored. Knowing the ancestry allows one to use ancestors to rescue the genetic variability in extreme cases in which some breeds are at risk of extinction.

Knowledge of the genetic composition of native breeds is essential for conservation and breeding programs of these genetic resources. This study is the first on the origin and genetic differentiation of the three Creole chicken breeds (CP, CAN, and PEL) from the northeast region of Brazil. Thus, our results will contribute directly to conservation of these chickens and encourage the conduction of new genetic studies in other states of Brazil.

### 4.2. Genetic Relationships between Brazilian and Exotic Chicken Breeds

Results of the first axis obtained in the PCoA indicate that exotic birds may have participated as ancestors during the formation of the Brazilian local chicken populations. The second axis indicates that most of the current Spanish breeds have a small contribution to the formation of the Brazilian chickens.

The matrix of Nei’s genetic distance shows that the Brazilian breeds studied have multiple origins. Chickens of the CP, CAN, and PEL breeds produce multicolored eggs, including eggs with bluish shells. This could explain the close relationship of these chickens with ARAU, in which hens produce only blue-colored eggs [28]. The genetic relationship among chicken breeds from Brazil and those from Portugal, Nigeria, and some Spanish genetic groups may be related to the colonization of Brazil. The phylogenetic relationships shown in the neighbor-net indicate that Brazilian chickens have multiple genetic origins. Nevertheless, these animals have some common ancestors which gave different percentages of contribution. CP was the only Brazilian breed which clustered together with a Spanish genetic group (Ibicenca).

The results of the analysis performed using STRUCTURE corroborates the results of the PCoA, which showed similar clusters for the two groups formed in K2. At this point, all Brazilian breeds were clustered together with birds from South America (Chile), Africa (Nigeria), and Europe (chickens from Portugal, Spain, and commercial Cornish strain). This result demonstrates the multiple ancestries of the Brazilian chickens. These birds showed low genetic similarity with most of the breeds from Spain, which indicates that this country had a small contribution to the formation of the chicken genetic groups from Northeastern Brazil.

It is important to note that in K5, the genetic composition of the CP breed was similar to that of the Nigerian chickens. This result corroborates the estimate of the matrix of Nei’s genetic distance, which shows that these two breeds are genetically closer. This relationship is probably due to the introduction of African animals in Brazil during the slavery period. Further, this could be explained by the fact that chickens were taken from Brazil to Africa after the abolition of slavery. In this period, several Africans returned to their home countries and some animals accompanied human migration [29].

In most situations, Delta K helps in the identification of the correct number of clusters; however, it should not be used exclusively [20]. We observed that from K6, some populations from different countries changed their genetic combinations. For the Brazilian breeds, the changes started in K7 and stabilized in K11. In this K, the CP chickens formed a single genetic group, whereas CAN and PEL clustered together and were different from all other groups. The genetic proximity of CAN and PEL is because both these breeds were formed in the same region of the Bahia state.

Note that from K10, none of the Brazilian breeds share genetic combinations with commercial strains. In K13 and K18, we confirm the genetic differentiation of CP from all other breeds in our study. The formation of these two possible groups indicates the genetic richness and exclusivity in the genetic composition of the Brazilian Creole chickens. Additionally, from K20, all breeds are defined in genetically different groups. In this case, the Portuguese chickens had the highest genetic migration. The results show that breeds from each country have a unique genetic structure, which demonstrates the genetic richness of the species *Gallus gallus* in these countries.

Our findings form the basis to justify and support the implementation of conservation programs of Brazilian local chicken breeds. Despite the genetic relationship with genetic groups from other countries, the Creole chickens from Brazil have unique genetic combinations of poultry from this country. Animals of these breeds are adapted to the edaphoclimatic conditions of the region and are spread throughout backyards of smallholders, serving as an example of multifunctionality of small-scale livestock production systems. Thus, promoting the raising of these birds in sustainable production systems will contribute to the development of livestock production in the northeast region of Brazil.

## 5. Conclusions

This study provides an overall outlook of the genetic diversity of chicken breeds from the northeast region of Brazil. Our findings highlight the efficiency of microsatellite markers for the study of the genetic characterization, differentiation, and relationship of chickens. In general, the populations of Canela-Preta, Caneluda do Catolé, and Peloco chickens are consolidated as defined breeds from a genetic point of view, because they have high genetic variability. In this study, we suggested that Brazilian chicken breeds are genetically stable, and there is high genetic difference between chickens from the Piauí and Bahia states and the other groups considered. The chickens from the northeast region of Brazil used in this study have an evolutionary relationship with breeds from Portugal, Nigeria, and Chile, as well as a weak relationship with Spanish chickens. Our results can be used in future strategies of genetic management of Brazilian free-range chickens as a basis for conservation programs, sustainable use, and genetic improvement through selection.

## Figures and Tables

**Table 1:** Chicken breeds used for assessment of identity and genetic relationships.

Breed	Abbreviation	Country/Region	Sample Size
Canela-Preta	CP	Brazil	40
Caneluda do Catolé	CAN	Brazil	30
Peloco	PEL	Brazil	30
Andaluza Azul	AAZ	Spain	50
Castellana Negra	CASN	Spain	50
Combatiente Español	CES	Spain	50
Extremeña Azul	EAZ	Spain	50
Ibicenca	IB	Spain	50
Mallorquina	MLL	Spain	50
Pita Pinta	PPA	Spain	50
Sureña	SUR	Spain	30
Utrerana Perdiz	UP	Spain	50
Leghorn	LEGH	Europe	40
Cornish	CORN	Europe	26
Araucana	ARAU	Chile	50
Nigerian chickens	NIG	Nigeria	50
Amarela	AM	Portugal	50
Branca	BR	Portugal	69
Preta Lusitânica	PL	Portugal	19
Pedrês Portuguesa	PP	Portugal	52
Total			886

**Table 2:** Mean and standard deviation (SD) for different estimates obtained at each locus for the three Brazilian breeds studied.

Genetic Group		N	Na	Ho	He	UHe	F	HWE (*p*-Value)
CP	Mean	39.8	4.960	0.617	0.618	0.626	−0.001	0.842
	SD		0.291	0.030	0.029	0.029	0.019	
CAN	Mean	29.6	5.040	0.642	0.634	0.645	−0.025	0.176
	SD		0.426	0.031	0.027	0.028	0.042	
PEL	Mean	29.6	4.960	0.646	0.620	0.630	−0.049	0.105
	SD		0.344	0.030	0.027	0.028	0.027	

CP, Canela-Preta; CAN, Caneluda do Catolé; PEL, Peloco; N, number of individuals; Na, mean number of alleles; Ho, observed heterozygosity; He, expected heterozygosity; UHe, expected heterozygosity with correction factor for sample size; F, Wright’s fixation index ([1 − (Ho/He)]); HWE, Hardy–Weinberg equilibrium test. Significance: *p* < 0.05.

**Table 3:** Statistics of the analysis of molecular variance (AMOVA) using 25 microsatellite loci in three Brazilian Creole chicken breeds.

Source of Variation	DF	SS	MS	Var. Comp.	%T	F (*p*-Value)
Between populations	2	110.817	55.480	0.714	8	FST = 0.082 (0.001) ^1^
Between individuals	97	800.858	8.257	0.234	3	FIT = 0.108 (0.001) ^2^
Within individuals	100	779.000	7.790	7.790	89	FIS = 0.029 (0.042) ^3^
Total	199	1690.775	0.027	8.738	100	

DF, degrees of freedom; SS, sum of squares; MS, mean square; Var. Comp., variance component; %T, percentage of total variance contributed by each component; ^1^ fixation index between nuclei; ^2^ fixation index between individuals; ^3^ fixation index within individuals.

## Supplementary Materials

The following are available online at https://www.mdpi.com/2076-2615/10/10/1857/s1, FigureS1: Map showing the geographical locations of Brazil, Portugal, and Spain, Figure S2: Graphical representation of the K values for the formation of clusters and analysis of population structure of 886 chickens representing five chicken groups based on 25 microsatellite markers, Table S1: Matrix of Nei’s genetic distance representing the 20 groups of chickens studied (18 native breeds and two commercial strains) based on 25 microsatellite markers.
