# Supplementary material for: Diversity and Genetic Relationship of Free-Range Chickens from the Northeast Region of Brazil
Source: Animals (Basel). 2020 Oct 12;10(10):1857. doi: 10.3390/ani10101857 (PMC7600294; doi:10.3390/ani10101857)
Supplement: Supplementary file 1 [file animals-10-01857-s001.pdf]

animals

Supplementary Materials: Diversity and Genetic Relationship of Free-Range Chickens from the Northeast Region of Brazil

Débora Araújo de Carvalho [1,*], Amparo Martínez Martínez [2,3], Inês Carolino [4,5], Maria Claudene Barros [6], María Esperanza Camacho Vallejo [7], Fátima Santos-Silva [4], Marcos Jacob de Oliveira Almeida [8], Nuno Carolino [4,5,9], Juan Vicente Delgado Bermejo [2] and José Lindenberg Rocha Sarmento [1,*]

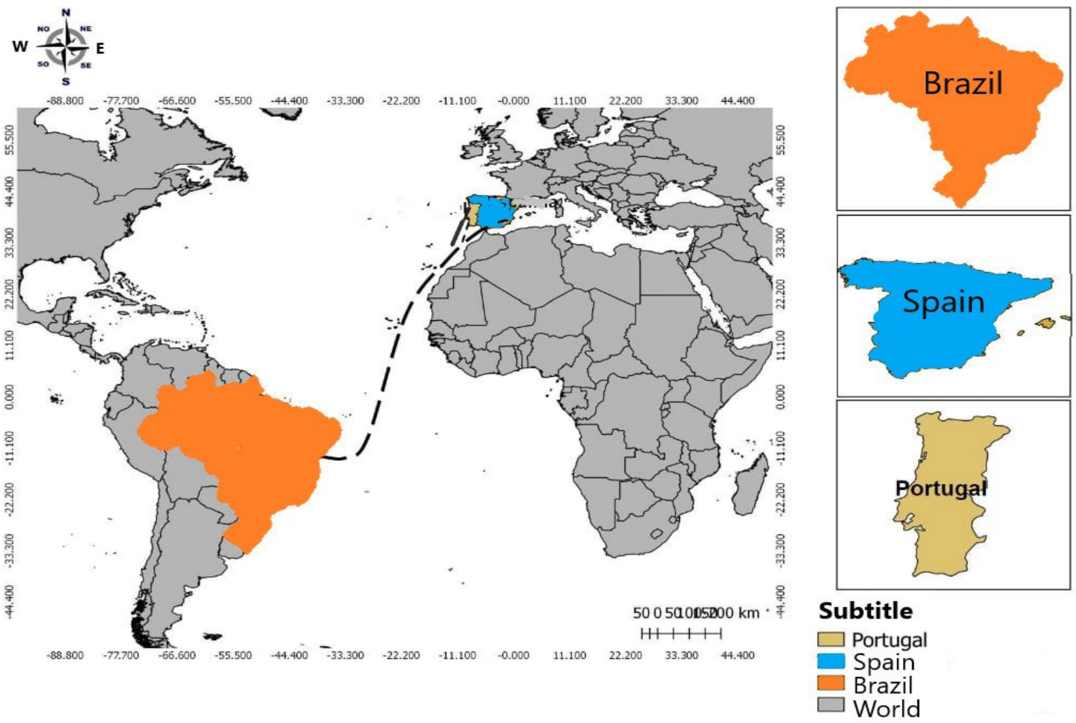

Figure S1. Map showing the geographical locations of Brazil, Portugal, and Spain.

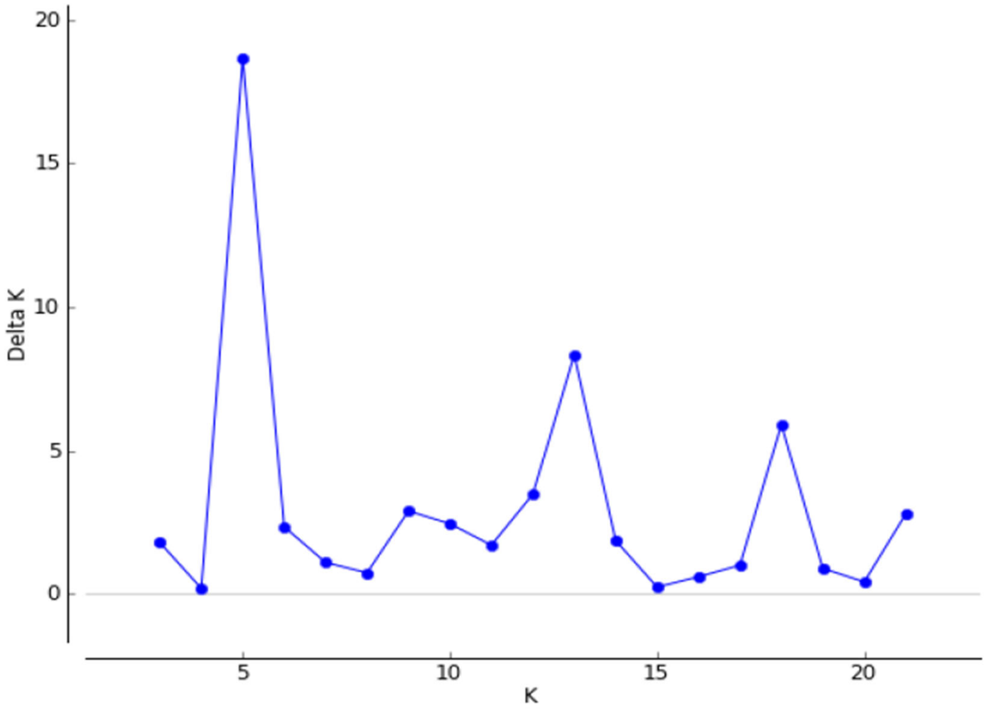

Figure S2. Graphical representation of the K values for the formation of clusters and analysis of population structure of 886 chickens representing five chicken groups based on 25 microsatellite markers.

Table S1. Matrix of Nei's genetic distance representing the 20 groups of chicken studied (18 native breeds and two commercial strains) based on 25 microsatellite markers.

Gen. G.	CP	CAN	PEL	AAZ	CASN	CES	EAZ	IB	MLL	PPA	SUR	UP	LEGH	CORN	ARAU	NIG	AM	BR	PLU
CAN	0.268	-																	
PEL	0.244	**0.063**	-																
AAZ	0.423	0.490	0.542	-															
CASN	0.263	0.263	0.270	0.175	-														
CES	0.336	0.412	0.410	0.225	0.113	-													
EAZ	0.293	0.271	0.259	0.370	0.196	0.313	-												
IB	0.222	0.233	0.233	0.412	0.262	0.393	0.199	-											
MLL	0.419	0.396	0.407	0.399	0.269	0.405	0.386	0.362	-										
PPA	0.314	0.295	0.294	0.358	0.327	0.388	0.345	0.301	0.435	-									
SUR	0.256	0.336	0.340	0.351	0.189	0.197	0.252	0.275	0.460	0.439	-								
UP	0.289	0.291	0.246	0.313	0.131	0.216	0.213	0.311	0.327	0.363	0.239	-							
LEGH	0.356	0.457	0.420	0.437	0.280	0.377	0.381	0.402	0.540	0.581	0.256	0.278	-						
CORN	0.271	0.224	0.240	0.457	0.237	0.367	0.303	0.249	0.343	0.308	0.308	0.258	0.397	-					
ARAU	0.242	**0.170**	0.191	0.332	0.194	0.321	0.161	0.159	0.316	0.214	0.235	0.237	0.336	0.213	-				
NIG	0.208	0.192	0.231	0.358	0.211	0.329	0.240	0.173	0.385	0.292	0.205	0.251	0.342	0.282	0.202	-			
AM	0.216	0.209	0.192	0.414	0.216	0.316	0.229	0.198	0.313	0.248	0.238	0.269	0.388	0.234	0.156	0.215	-		
BR	0.334	0.222	0.229	0.506	0.285	0.369	0.273	0.269	0.430	0.257	0.278	0.299	0.480	0.278	0.191	0.264	0.162	-	
PL	0.276	0.198	**0.156**	0.540	0.266	0.429	0.251	0.296	0.384	0.317	0.325	0.283	0.457	0.269	0.227	0.273	0.197	0.251	-
PP	0.242	0.198	0.174	0.501	0.250	0.428	0.249	0.170	0.376	0.287	0.286	0.292	0.348	0.241	0.202	0.192	0.153	0.200	0.163
